# Factors Influencing the Dementia-Preventive Behaviors among Middle-Aged Persons with Chronic Diseases in Korea

**DOI:** 10.3390/ijerph18062936

**Published:** 2021-03-13

**Authors:** Jisung Park, Juh Hyun Shin

**Affiliations:** 1 Department of Nursing, Seoul Women’s College of Nursing, Seoul 03617, Korea; 2 Science & Ewha Research Institute of Nursing Science, College of Nursing, Ewha Womans University, Seoul 120750, Korea; juhshin@ewha.ac.kr

**Keywords:** middle-aged, dementia, health literacy, health behavior, fear of dementia, locus of control

## Abstract

The purpose of this study was to investigate the influence of dementia literacy, internal health locus of control, and fear of dementia on dementia-preventive behaviors among middle-aged people with chronic diseases. The participants were middle-aged individuals with chronic diseases who had been taking medications for at least three months, recruited using convenience sampling. A total of 123 participants were recruited between 13 and 31 March 2020, using self-reported questionnaires. Data were then analyzed through independent t-test, one-way ANOVA, Pearson’s correlation coefficient, and multiple linear regression using the SPSS/WIN 25.0. The results showed that health condition perceived as healthy and dementia literacy were the leading factors influencing dementia-preventive behaviors. These variables showed a 16% explanatory power for dementia-preventive behaviors. Therefore, when the participants’ perceived health condition was healthy and the dementia literacy score was higher, the level of dementia-preventive behaviors was also higher. It is necessary to develop educational programs to increase dementia literacy as a major variable in dementia-preventive behaviors, and further research on its efficacy should be conducted. When providing dementia-preventive education programs to middle-aged people who have been exposed to chronic diseases, it is necessary to consider their level of perceived health condition and dementia literacy.

## 1. Introduction

The number of people with dementia worldwide is on the rise, with 46.8 million reported in 2015, increasing to 74.7 million in 2030 and 131.5 million in 2050 [1]. Among those eligible for long-term care insurance for the elderly in Korea, the number of middle-aged dementia patients under the age of 65 has more than doubled from 24,387 in 2010 to 49,751 in 2018 [2]. Thus far, no curative treatment has been developed for dementia, and so a preventive approach is more important than a therapeutic approach [3,4]. Depression, smoking, and hearing loss, along with hypertension, diabetes, and hyperlipidemia, among middle-aged individuals are important risk factors for dementia in old age [1]. In particular, middle-aged individuals with high blood pressure, diabetes, and hyperlipidemia are at a high risk of developing dementia [5]; therefore, attention needs to be paid to dementia prevention and management for middle-aged individuals who have been exposed to chronic diseases. Increasing the implementation of dementia-preventive behaviors, such as the thorough management of blood pressure, blood sugar and triglyceride, and avoiding a lack of exercise, smoking, drinking and social isolation from middle age onwards, would minimize the likelihood of developing dementia [6]. However, middle-aged people are less interested in preventing dementia because they perceive dementia negatively, as an inevitable phenomenon in the aging process [6,7]. Research is therefore needed to identify the factors that affect dementia-preventive behaviors in middle-aged people with chronic diseases.

As the onset of dementia increases in frequency, dementia literacy has become increasingly important [8,9]. Dementia literacy refers to the ability to acquire, understand, evaluate and use information on dementia-related prevention, management and early treatment [10]. A higher concept of dementia literacy, which is health literacy, has been shown to increase the implementation of health behaviors among patients with chronic diseases [11,12]. Despite the high possibility of developing dementia, middle-aged individuals who have been exposed to chronic diseases lack dementia literacy [13,14]. Since increasing dementia literacy reduces the risk of developing dementia, enables early diagnosis and management [9] and enhances individual dementia response capabilities [15], this study considered dementia literacy to be a variable related to dementia-preventive behaviors.

The principle of the internal health locus of control tends to be based on the belief that one’s actions will affect the outcome [16]. The higher the internal health locus of control, the higher the dementia-preventive behaviors, and people whose internal health locus of control is high tend to actively engage in healthy behaviors [17,18]. Thus, it is important to focus on the internal health locus of control as a variable linked to dementia-preventive behaviors.

Throughout life, middle-aged people dread dementia as the disease they would most like to avoid [19]. Fear of dementia is an emotional response caused by recognizing the fear of developing dementia [20]. Thirty-eight percent of middle-aged people are worried about the onset of dementia and three percent are very worried [6], but dementia-preventive behaviors are rather lower in this age group than in older people [14]. Since the appropriate fear of dementia leads to real attention that can help to prevent dementia [21], identifying the level of fear of dementia among middle-aged individuals who have been exposed to chronic diseases can be considered an important variable in explaining dementia-preventive behaviors.

This study attempted to identify the factors influencing dementia-preventive behaviors among middle-aged individuals with chronic diseases. The purpose of this study was to check the level of dementia literacy, internal health locus of control, fear of dementia and dementia-preventive behaviors; to identify the correlation of variables and differences in dementia-preventive behaviors according to general characteristics; and to determine the degree of influence of related variables. Moreover, the outcomes of the study can contribute to providing basic data for developing nursing interventions and systematic dementia-preventive programs that can effectively promote dementia-preventive behaviors.

## 2. Materials and Methods

### 2.1. Study Design

This study is a descriptive survey conducted to identify the factors influencing dementia-preventive behaviors among middle-aged people who have been exposed to chronic diseases. 

### 2.2. Participants 

The participants were middle-aged men and women aged 45 to 65 years living in Seoul, Korea, who were diagnosed with one or more of the following chronic diseases: high blood pressure, diabetes and hyperlipidemia. Convenience sampling was used in this study. The advertisement flyers for recruiting study participants were distributed in two apartment community centers and companies. Those who voluntarily wanted to participate were asked to use the researchers’ contact information. The criteria for inclusion were middle-aged individuals who were taking regular medication for more than 3 months after being diagnosed with chronic diseases: high blood pressure, diabetes, or hyperlipidemia. In addition, they had to be able to understand the content of the questionnaires and agree to participate in the survey. The exclusion criteria were individuals with cognitive disorders or other psychiatric diagnosis, such as dementia or mild cognitive impairment, as well as, persons who were cognitively impaired due to brain damage (head trauma, brain tumor). The number of participants was calculated before recruiting study participants, using the G*power 3.1 program. The criteria were based on prior studies of dementia-preventive behaviors [3,4,22], a medium effect size of 0.15, a significance level of 0.05 and a power of 0.95. For multiple regression analysis, the number of participants needed was 119. Considering the dropout rate, a total of 130 questionnaires were distributed and 123 responses were used for the analysis (7 incomplete questionnaires were excluded).

### 2.3. Measures

This study structured questionnaires consisting of 20 general characteristics, 16 dementia literacy measures, 6 internal health locus of control measures, 30 fear of dementia measures, and 15 measures of dementia-preventive behaviors, for a total of 87 questions.

#### 2.3.1. Dementia Literacy

The dementia health literacy scale used in this study was developed by Kim et al. [23]. It consists of four sub-regions—prevention, risk factors, treatment information, and care services—with a total of 16 questions. Each item was measured on a 5-point Likert scale ranging from 1 to 5, and the lower the score, the higher the level of dementia literacy. During tool development [23], the Cronbach’s α was 0.89, and in this study, the Cronbach’s α was 0.97.

#### 2.3.2. Internal Health Locus of Control 

The internal health locus of control scale used in this study was developed by Wallston et al. [16] and redeveloped by Park [24] for use in a Korean setting. It consists of six questions and is measured on a 5-point Likert scale. The higher the score, the higher the level of internal health locus of control. During tool development, the Cronbach’s α was 0.81, and in this study, the Cronbach’s α was 0.86.

#### 2.3.3. Fear of Dementia

The fear of dementia scale used in this study was developed by French et al. [20]. It was translated into Korean as the Korean version of the fear of Alzheimer’s disease scale (FADS) by Moon et al. [25]. It consists of three sub-regions: general fear (17 questions), physical symptoms (8 questions) and tragic attitudes (5 questions). Each item was measured on a 5-point Likert scale ranging from a minimum of zero to a maximum of four. The higher the score, the greater the fear of dementia. During tool development [20], the Cronbach’s α was 0.94, and in this study, the Cronbach’s α was 0.95.

#### 2.3.4. Dementia-Preventive Behaviors

The dementia-preventive behaviors scale used in this study was developed by Lim et al. [26], which is a “dementia prevention rules: recommendation, prohibition, conductive” scale. It consists of three sub-regions: recommended rules (five questions), prohibited rules (five questions), and conductive rules (five questions). Each item was measured on a 5-point Likert scale; the higher the score, the higher the level of dementia-preventive behaviors. During tool development [26], the Cronbach’s α was 0.77, and in this study, the Cronbach’s α was 0.71.

### 2.4. Data Collection and Ethical Considerations

After approval was obtained from the E University Institutional Review Board (ewha-202002-0025-02), this study was conducted between 13 and 31 March 2020, using self-reported questionnaires. Data were collected using a convenience sampling method for middle-aged men and women who had been taking regular medication for more than three months after being diagnosed with high blood pressure, diabetes, or hyperlipidemia. The researcher explained the purpose and necessity of the study, that participation could be withdrawn at any time, the anonymous nature of the study, and that the contents of the survey would only be used for research purposes. The data collection was conducted in cafes, parks, companies and homes in Seoul. After giving written consent for participation in the study, the participants completed the questionnaires themselves, taking an average time of 15–20 min. 

### 2.5. Data Analysis

The collected data were analyzed using SPSS/WIN 25.0 (IBM Corp., Armonk, NY, USA). To identify the general characteristics of the participants, we calculated the frequency, percentage, mean, and standard deviation. The level of participants’ dementia literacy, internal health locus of control, fear of dementia, and dementia-preventive behaviors were calculated using frequency, percentage, mean, and standard deviation. Differences in dementia-preventive behaviors were analyzed by independent t-test and one-way ANOVA, and post-analysis was verified by Scheffé’s post-hoc test. The relationship between dementia literacy, internal health locus of control, fear of dementia and dementia-preventive behaviors was analyzed using Pearson’s correlation coefficient. Factors influencing dementia-preventive behaviors were analyzed using multiple linear regression analysis.

## 3. Results

### 3.1. General Characteristics of Participants

Table 1 presents the participants’ demographic characteristics. Of a total of 123 participants, some participants had experience in dementia family care for now-deceased family members.

### 3.2. Participants’ Degree of Dementia Literacy, Internal Health Locus of Control, Fear of Dementia and Dementia-Preventive Behaviors

Dementia literacy was 54.75 ± 12.98 out of 80 points, the internal health locus of control score was 25.46 ± 3.49 out of 30 points, and the fear of dementia score was 42.98 ± 21.48 out of 120 points. The dementia-preventive behaviors score was 43.58 ± 6.63 out of 65 points.

### 3.3. Differences in Dementia-Preventive Behaviors According to the General Characteristics

There were statistically significant differences in age (t = −2.52, *p* = 0.013), perceived health conditions (F = 10.20, *p* < 0.001) and dementia knowledge level (F = 2.81, *p* = 0.042). Scheffé’s post-hoc test showed significantly higher scores for participants who were perceived as healthy than for those with normal or poor health, and for older participants, dementia-preventive behaviors were higher (Table 2).

### 3.4. Correlation between Dementia Literacy, Internal Health Locus of Control, Fear of Dementia, and Dementia-Preventive Behaviors

Dementia literacy showed a significantly positive correlation with dementia-preventive behaviors (r = 0.25, *p* = 0.006). This indicates that the higher the dementia literacy, the greater the incidence of dementia-preventive behaviors. The internal health locus of control (r = 0.12, *p* = 0.197) and fear of dementia (r= −0.15, *p* = 0.110) were not significantly correlated with dementia-preventive behaviors.

### 3.5. Factors Influencing the Dementia-Preventive Behaviors

Multiple regression analysis was conducted among the general characteristics that showed statistically significant differences in dementia-preventive behaviors. The variables included were perceived health conditions, dementia literacy, internal health locus of control and fear of dementia as independent variables. The perceived health conditions were analyzed by converting them into dummy variables. The multiple regression model was statistically significant (F = 5.78, *p* < 0.001). The Durbin–Watson statistic was 2.07, approximating 2, which indicated that there was no problem with the assumption of independence of the residuals. The variance inflation factor (VIF) was less than 10 and multicollinearity was found to be no problem, indicating that the data used in the study were appropriate for regression analysis.

As a result of validating the significance of the regression coefficient, a healthy perceived health condition (β = 0.50, *p* = 0.004) and dementia literacy (β = 0.21, *p* = 0.013) influenced dementia-preventive behaviors. These variables showed a 16% explanatory power for dementia-preventive behaviors. Therefore, participants who perceived themselves as healthy and had a higher level of dementia literacy also exhibited greater dementia-preventive behaviors (Table 3).

## 4. Discussion

This study attempted to identify the degree of dementia literacy, internal health locus of control, fear of dementia and dementia-preventive behaviors; identify the correlation of variables and differences in dementia-preventive behaviors according to general characteristics; and determine the degree of variables’ impact on dementia-preventive behaviors.

The results indicated that when a participant’s health condition was perceived as healthy, dementia-preventive behaviors were evaluated as increasing. This corresponds with the results of previous studies on older people living alone [17] and middle-aged people in their 40s and 60s [22]. The data also supported a previous study [27] of middle-aged people that showed the higher the subjective health status, the higher their health-promoting behavior.

As a result of this study, dementia-preventive behaviors reportedly strengthened as dementia literacy increased. Due to the limited research on the relationship between dementia-preventive behaviors and dementia literacy, a comparative perspective was gained from the higher concept of health literacy. In the light of studies [11,12] showing that health literacy is a major influencing factor in the implementation of health behaviors, dementia literacy can be inferred as a major factor in dementia-preventive behaviors. With an aging society and the increasing complexity of healthcare systems, dementia literacy is becoming increasingly important [8,9]. When the participants’ perceived health condition was healthy and dementia literacy was high, dementia-preventive behaviors were high as well. Individual dementia prevention education programs should therefore be provided to middle-aged people who are exposed to chronic diseases after evaluating their perceived health conditions and level of dementia literacy.

Meanwhile, the Korean Ministry of Health and Welfare [28] operates a dementia safety center to facilitate dementia prevention management projects, dementia perception improvement, education and promotion. In addition, there is a need to designate middle-aged individuals with chronic diseases as a category at risk of dementia, in order to expand opportunities for access to dementia prevention content, strengthening practice and seeking various promotional measures.

According to studies of ordinary middle-aged coronary artery disease patients, college students, and non-smokers, a higher internal health locus of control contributes to higher health-promoting behaviors, better control and management of the environment, and higher efficiency in the acquisition of information [29,30,31,32]. As a result, they become more interested in their health condition by participating in healthy activities and treatment [29,30,31,32]. However, in this study, the internal health locus of control was found to have no explanatory power as a predictor of dementia-preventive behaviors in middle-aged individuals with chronic diseases. These results were different from those of previous studies [17,33], which showed that the higher the internal health locus of control, the higher the health-promoting behaviors and dementia-preventive behaviors. It is difficult to compare the results of this study with previous ones because they were conducted on elderly and coronary artery disease patients. In addition, since prior research on dementia-preventive behaviors studying internal health locus of control is extremely rare, it is difficult to compare outcomes. It is therefore recommended that follow-up studies are conducted to gain a better understanding of the trends. This study also found that fear of dementia was not a predictor of dementia-preventive behaviors among middle-aged individuals with chronic diseases. It differed from a previous study [22], which found that the higher the fear of dementia, the lower the dementia-preventive behaviors. According to prior research, if fear of dementia is excessive, it can negatively affect dementia-preventive behaviors due to negative perceptions such as health concerns and neuroticism [6,34]. On the other hand, an appropriate fear of dementia can help establish a healthy dementia-preventive lifestyle by creating a realistic interest in dementia [21,35]. As such, the results of the dementia fear variable differed depending on the prior studies. A possible explanation for the difference between the results of the current and previous research is that the participants in this study were middle-aged, not elderly, but people with chronic diseases. In this population group, fear of dementia tends to be lower (42.98 ± 21.48 out of 120 points), so it is believed that it was not included as an influencing factor of dementia-preventive behaviors. Further research on the role of fear of dementia as a variable among the influencing factors of dementia-preventive behaviors is needed.

The results of this study showed that perceived health condition and dementia literacy are the main factors influencing dementia-preventive behaviors among middle-aged people with chronic diseases. Among these factors, the most influential factor was when a participant’s perceived health condition was healthy. A limitation of this study is that it was conducted by convenience sampling middle-aged men and women with chronic diseases in a small setting. The number of participants was biased toward men; thus, the study results may not be generalizable to all middle-aged persons with chronic diseases.

## 5. Conclusions

This study aimed to identify basic information to be used for the development of nursing interventions and systematic dementia-prevention programs to promote dementia-preventive behaviors in middle-aged people who have been exposed to chronic diseases. As a result of this study, it was reported that perceived health condition as healthy and dementia literacy were the leading factors influencing dementia-preventive behaviors, with a 16% explanatory power. Therefore, when the participants’ perceived health condition was healthy and their dementia literacy score was higher, the incidence of dementia-preventive behaviors was higher too.

Based on the above results, it is firstly proposed that more research on the development of individualized education programs is needed, especially on the role of perceived health conditions and dementia literacy as major variables in dementia-preventive behaviors. Secondly, additional research considering the size of the residential district and the characteristics of various participants is needed. This will overcome the limited generalizability of this study, which tended to be biased toward men. Thirdly, most dementia education is currently aimed at senior citizens aged 65 or older; therefore, nursing intervention is needed to improve dementia prevention for middle-aged people who are exposed to chronic diseases.

## Figures and Tables

**Table 1:** General characteristics of middle-aged persons with chronic diseases (*N* = 123).

Characteristics	Categories	n (%) or M ± SD	Characteristics	Categories	n (%)
Gender	Men	85 (69.1)	Dementia in the family	Yes	23 (18.7)
	Women	38 (30.9)		No	100 (81.3)
Age (year)	-	56.35 ± 5.87	Dementia family care experience	Yes	29 (23.6)
				No	94 (76.4)
Marriage status	Married	114 (92.7)	Whether they have chronic disease patients in their families	Yes	96 (78.0)
	etc. (unmarried, bereavement)	9 (7.3)		No	27 (22.0)
Level of education	≤High school	12 (9.8)	Degree of dementia interest	Very interested	19 (15.4)
	College	73 (59.3)		Interested	46 (37.4)
	≥Graduate school	38 (30.9)		Middle	54 (43.9)
				Not interested	4 (3.3)
Average monthly income (10,000 won)	≤150	14 (11.4)	Dementia knowledge level	Know very well	7 (5.7)
	151–300	20 (16.3)		Know well	18 (14.6)
	301–450	15 (12.2)		Middle	73 (59.4)
	≥451	74 (60.1)		Know little	25 (20.3)
Current disease ^†^	Hypertension	80 (43.5)	The degree of awareness of the possibility of getting dementia	High	22 (17.9)
	Diabetes	33 (17.9)			
	Hyperlipidemia	65 (35.3)		Middle	71 (57.7)
	Cerebrovascular disease	1 (0.6)		Low	30 (24.4)
	Heart disease	5 (2.7)			
The duration of taking chronic diseases medication (year)	-	6.37 ± 5.80	Dementia education experience	Yes	12 (9.8)
				No	111 (90.2)
Perceived health conditions	Healthy	49 (39.8)	Perceived the necessity of dementia education	Yes	110 (89.4)
	Middle	66 (53.7)			
	Not healthy	8 (6.5)		No	13 (10.6)
Dementia information access path ^†^	Newspaper	21 (12.7)	Whether exposed to dementia information	Yes	69 (56.1)
	Broadcasting	48 (29.1)			
	Internet	32 (19.4)			
	Magazine	2 (1.2)			
	Educational materials	18 (10.9)		No	54 (43.9)
	Family or Relatives	27 (16.4)			
	Friends or Colleagues	17 (10.3)			

M = mean; SD = standard deviation; ^†^ multiple response question.

**Table 2:** The differences in dementia-preventive behaviors according to general characteristics of middle-aged persons with chronic diseases (*N* = 123).

Characteristics	Categories	Dementia-Preventive Behaviors	
		M ± SD	t/F (*p*)
			Scheffé
Gender	Men	3.30 ± 0.53	−1.63
	Women	3.46 ± 0.45	(0.105)
Age	45–54	3.19 ± 0.41	−2.52
(year)	55–65	3.43 ± 0.54	(0.013)
The duration of taking chronic disease medication	3 months–<1 y	3.23 ± 0.50	0.23
	1–3 y	3.40 ± 0.59	(0.876)
	3–<6 y	3.34 ± 0.50	
	≥6 y	3.35 ± 0.47	
Perceived health conditions	Healthy	3.58 ± 0.43	10.2
	Normal	3.22 ± 0.52	(<0.001)
	Poor	3.04 ± 0.36	
Whether they have chronic disease patients in their families	Yes	3.35 ± 0.49	−0.14
	No	3.36 ± 0.58	(0.885)
Dementia level of knowledge	Know very well	3.77 ± 0.48	2.81
	Know well	3.30 ± 0.46	(0.042)
	Middle	3.38 ± 0.52	
	Know little	3.18 ± 0.45	
The degree of awareness of the possibility of getting dementia	High	3.22 ± 0.45	1.93
	Middle	3.18 ± 0.37	(0.110)
	Low	3.08 ± 0.46	
Dementia education experience	Yes	3.49 ± 0.45	1.01
	No	3.34 ± 0.52	(0.314)
Perceived the necessity of dementia education	Yes	3.37 ± 0.51	1.26
	No	3.18 ± 0.46	(0.209)
Whether exposed to dementia information	Yes	3.42 ± 0.49	−1.66
	No	3.27 ± 0.53	(0.099)

SD = standard deviation.

**Table 3:** Factors influencing dementia-preventive behaviors of middle-aged persons with chronic diseases (*N* = 123).

Variables		B	SE	β	t	*p*	TOL	VIF
(Constant)		2.37	0.41	-	5.83	<0.001		
Perceived health conditions	(ref: Not healthy)							
	Healthy	0.52	0.18	0.50	2.90	0.004	0.23	4.35
	Middle	0.21	0.18	0.20	1.17	0.244	0.23	4.34
Dementia Literacy		0.13	0.05	0.21	2.53	0.013	0.99	1.01
Internal Health Locus of Control		0.07	0.07	0.07	0.87	0.385	0.95	1.05
Fear of Dementia		−0.04	0.06	−0.06	−0.67	0.503	0.92	1.09
R² = 0.20, Adj. R² = 0.16 Durbin–Watson = 2.07, F (*p*) = 5.78 (<0.001)								

SE = standard error; TOL = tolerance; VIF = variance inflation factor; ref = reference.

## Data Availability

The data used and/or analyzed during this study are available from the corresponding author on request.

## References

[1] Prince M., Wimo A., Guerchet M., Ali G.C., Wu Y.T., Prina M. (2015). World Alzheimer’s Report 2015: The Global impact of Dementia.

[2] Statistics Korea (2019). Status of Senior Diseases among Applicants under the Age of 65 by City and Province. Press Release. http://kosis.kr/statHtml/statHtml.do?orgId=350&tblId=DT_35006_N006&vw_cd=MT_ZTITLE&list_id=350_35006_A002&seqNo=&lang_mode=ko&language=kor&obj_var_id=&itm_id=&conn_path=MT_ZTITLE.

[3] Lee Y.R. (2020). A study on dementia attitudes and preventive behaviors in dementia care workers. JLCCI.

[4] Choi H.J., Kim J.S. (2019). A Study on the Dementia Policy Recognition, Dementia Knowledge, Dementia Attitude and Dementia Prevention Behaviors of Middle-Aged.

[5] Horder H., Johansson L., Guo X. (2018). Midlife cardiovascular fitness and dementia: A 44-year longitudinal population study in women. Neurology.

[6] Bowen C.E., Kessler E.M., Segler J. (2019). Dementia worry in middle-aged and older adults in Germany: Sociodemographic, health-related and psychological correlates. Eur. J. Ageing.

[7] NatCen Social Research. https://www.bsa.natcen.ac.uk/latest-report/british-social-attitudes-33/dementia.aspx.

[8] Rostamzadeh A., Stapels J., Genske A., Haidl T., Jünger S., Seves M. (2020). Health literacy in individuals at risk for alzheimer’s dementia: A systematic review. JPAD.

[9] Smith B.J., Ali S., Quach H. (2014). Public knowledge and beliefs about dementia risk reduction: A national survey of Australians. BMC Public Health.

[10] World Health Organization (2012). Dementia: A Public Health Priority. Institutional Repository for Information Sharing. https://who.int/iris/bitstream/handle/10665/75263/9789241564458_eng.pdf?sequence=1&isAllowed=y.

[11] Oh J.H., Park E.N. (2017). The impact of health literacy on self-care behaviors among hypertensive elderly. Korean J. Hep..

[12] Baron-Epel O., Levin-Zamir D., Cohen V., Elhayany A. (2019). Internal locus of control, health literacy and health, an Israeli cultural perspective. Health Promot. Int..

[13] Heger I., Deckers K., Van Boxtel M., De Vugt M., Hajema K., Verhey F. (2019). Dementia awareness and risk perception in middle-aged and older individuals: Baseline results of the MijnBreincoach survey on the association between lifestyle and brain health. BMC Public Health.

[14] Livingston G., Sommerlad A., Orgeta V., Costafreda S.G., Huntley J., Ames D. (2017). Dementia prevention, intervention, and care. Lancet.

[15] Kim S.B. (2018). A Study of Healthcare & Social Welfare Workers’ Dementia Literacy. Master’s Thesis.

[16] Wallston K.A., Wallston B.S., DeVellis R. (1978). Development of the multidimensional health locus of control scales. Health Educ. Monogr..

[17] Kang N.G., Yoo M.S., Song M.S., You M.A. (2015). The effect of knowledge on dementia and internal health locus of control on dementia preventive behaviors among the Korean older people living alone. J. Health Inform. Stat..

[18] Cha N.H. (2013). The relationships between stress and health locus of control in nursing college students. J. East West Nurs. Res..

[19] Alzheimer’s Association (2014). 2014 Alzheimer’s disease facts and figures. Alzheimer’s Dement..

[20] French S.L., Floyd M., Wilkins S., Osato S. (2012). The fear of alzheimer’s disease scale: A new measure designed to assess anticipatory dementia in older adults. Int. J. Geriatr. Psychiatry.

[21] Smyth C. (2015). Dementia Clinics are Swamped by Worried Well. The Times: England. https://www.thetimes.co.uk/article/dementia-clinics-are-swamped-by-worried-well-8slv78sq9j5.

[22] Park M., Oh D., Moon H. (2018). A study on dementia related attitudes of the middle aged and their dementia preventive behaviors. J. Korea Contents Assoc..

[23] Kim Y., Lee H., Oh Y., Kang E. (2017). Reliability and validity of the dementia health literacy scale: A pilot study. Innov. Aging.

[24] Park B.Y. (2008). Factors Influencing the Health Promotion Behaviors among Middle-Aged Women. Master’s Thesis.

[25] Moon Y.S., Kim H.J., Choi H., Oh S.I., Han S.H. (2014). Validity of the Korean version of the fear of alzheimer’s disease scale for the assessment of anticipatory dementia. JKMS.

[26] Lim K.C., Kim J.Y., Kim M.S. (2018). Development and Verification of the “Dementia Prevention Rules, Recommendation, Prohibition, Conductive” Scale for the Elderly in Korea.

[27] Kim J., Kwon M., Jung S. (2017). The influence of health locus of control, social support, and self-efficacy on health promoting behavior in middle-aged adults. JKAIS.

[28] Ministry of Health and Welfare 2020 Dementia Policy Guide. http://www.mohw.go.kr/react/jb/sjb030301vw.jsp?PAR_MENU_ID=03&MENU_ID=032901&CONT_SEQ=352660&page=1.

[29] Kim S.H., Bae K.R. (2013). The effects of health control behavior of middle-aged people on the health promotion behavior. J. K AHW.

[30] Zou H., Tian Q., Chen Y., Cheng C., Fan X. (2017). Coping styles mediate the relationship between self-esteem, health locus of control, and health-promoting behavior in Chinese patients with coronary heart disease. JCN.

[31] Acikgoz S., Kitis Y. (2017). Relationship between healthy lifestyle behaviors and health locus of control and health-specific self-efficacy in university students. Jpn. J. Nurs. Sci..

[32] Holt C.L., Roth D.L., Huang J., Clark E.M. (2015). Gender differences in the roles of religion and locus of control on alcohol use and smoking among African Americans. JSAD.

[33] Shin N., Kang Y. (2015). The relationships among health locus of control and resilience, social support and health promoting behavior in patients with newly diagnosed coronary artery diseases. KJAN.

[34] Petkus A.J., Reynolds C.A., Wetherell J.L., Kremen W.S., Pedersen N.L., Gatz M. (2016). Anxiety is associated with increased risk of dementia in older Swedish twins. Alzheimer’s Dement..

[35] Kim J.S., Kim E.H., An M. (2016). Experience of dementia-related anxiety in middle-aged female caregivers for family members with dementia: A phenomenological study. Asian Nurs. Res..

